# Therapeutical and Pharmaceutical Notes

**Published:** 1901-06

**Authors:** W. J. Martin

**Affiliations:** Kankakee, Ill.


### THERAPEUTICAL AND PHARMACEUTICAL NOTES.

Under the Direction of W. J. Martin, M.D.C.,

KANKAKEE, ILL.

---

Median Neurectomy. In the *Veterinary Journal* for March, 1901, Prof. F. Hobday sums up his experience of the operation of median neurectomy extending over a period of five years. During this time he performed the operation on 500 cases; of this number the history of 100 cases had been traced up to the date of his report. His opinion is that the results of the operation have been excellent, though many of the cases operated on were old horses of the cab and cart variety, whose lameness had been long subjected to the classical treatment without avail previous to the operation. He thinks that section of the median nerve has a particular advantage over the double plantar operation, insomuch that its influence extends over a larger area, while at the same time there is less danger of sloughing of the foot owing to the nerve-supply which is still transmitted to the external plantar by the ulnar nerve. He states that according to French and German statistics about 2 per cent. is the average of sloughing of the foot after median neurectomy. In Prof. Hobday's operations in the first 100 cases but one case of gelatinous degeneration of the foot occurred, while in the whole 500 cases but two cases of sloughing of the foot took place. He also states that degeneration and rupture of the flexor tendons, which is often supposed to be a sequel of this operation, has not occurred in any of his operations. He thinks that in many of the cases operated on no interference with the action of the limb is noticed, and in many of the cases operated on for osseous exostosis there was a marked subsidence of the enlargement. He regards the dangers in connection with operation very slight, providing the operator pays strict attention to cleanliness and antiseptic precautions, such as shaving off the hair, washing with ether or soft soap the field of operation. In regard to puncture of the large artery or vein during operation he attaches no special importance, aside from the difficulty encountered from having the seat of operation obscured with blood. He states that he has seen the vein opened many times and even cut across without any permanent injury. This applies also to the artery, which he states he has seen cut across in mistake for the nerve on four separate occasions without any material injury beyond that the leg remained swollen for a week or two and the toe was dragged for a while, but in no instance did anything more serious occur. He recommends that the operation should never be performed unless chloroform or some local anæsthetic be used, both from a humane stand-point and also that it makes the operation easier for the operator. He finds that neuromas are of frequent occurrence after the operation, and states that such are usually very painful and recommends their removal, after which the animal generally goes sound again.

Prof. Hobday recommends the operation "for old-standing lameness, when due to splints, exostosis on the inside of the leg, chronically sprained, thickened and painful perforans or perforatus tendons, or cases of that kind which cause pain by pressing on the adjacent nerve structures. After all other known methods of treatment have failed median neurectomy is the operation which will be the most likely to give the animal a new lease of life and usefulness."

For diseases below the fetlock joint, such as navicular, ringbone, sidebone, etc., there appears to be no advantage whatever over ordinary internal plantar neurectomy, and we have the disadvantage of having unnecessarily deprived a large part of the limb of its nerve-supply.

In the treatment, however, of that form of exostosis which is so commonly met with on the inside of the leg of cab horses, division of the median nerve has given better results than division merely of the internal plantar. I have thought that probably this has been due to undetected complications in the region of the cannon bone. When mechanical interference has not given rise to a stiff joint, median neurectomy has given excellent results when the actual cautery has failed.

Treatment of Lameness by the Subcutaneous Injection of Essence of Turpentine. In an address delivered before the Central Society of Veterinary Medicine of France, M. Cagny recommends the subcutaneous injection of essence of turpentine in the treatment of chronic lameness of the fore extremities, more especially those of race-horses. His method of procedure is as follows: "Injections being always painful, especially in the case of nervous animals, it is important to put the animal on a cooling diet for at least a fortnight beforehand. He uses rectified essence of turpentine, but in order to assure asepsis and diminish pain he adds to ten grammes of essence about one gramme of a solution of guaiacol alcohol at 96°—a solution of 10 per cent. About the middle of the shoulder he makes four injections of one gramme each, almost on a horizontal line; two in front of the edge of the scapula and two behind. He also makes four injections on the two shoulders even for a lameness on the one side. The horse must then be left at liberty in the loose box with plenty of good litter. One must not be frightened at the symptoms which follow. Some horses are affected with a kind of vertigo, and this may go on for several hours; others refuse to eat and drink for more than twenty-four hours. On the following day the shoulders are swollen, hot, and painful to the touch, the swelling increases and descends to the elbows, movement becomes difficult, and the horse remains motionless, but gradually the œdema descends and movement becomes less painful; it becomes once more very difficult for twenty-four hours, when the infiltration reaches the knees. There are two important points: leave the animals alone whatever the symptoms of vertigo may be, and do not try to calm them or walk them about, but let them have full liberty in a wide space filled with straw. Afterward, however severe the œdema and however great the difficulty of movement may be, do not try puncture or friction or fomentations; do not touch the shoulders, even if you think you notice fluctuation. About the fourth week the horse may be walked up and down or set at liberty for a few hours in a meadow. Gradually movement becomes easier, but the cure is not complete until two months have passed."

For strained tendons M. Cagny makes only two injections of one gramme below the knee—one on the internal aspect, the other on the external, between the suspensory ligaments and flexor tendons. As there are only two injections, the symptoms are less accentuated, but often through one of the injections there is a discharge of serous liquid resembling pus. In this case he recommends fomentations with a sponge and cresylated water. The cause is probably the greater density of the cellular tissues, but the subcutaneous infiltration persists for several months. It disappears only slowly and with graduated effort. Judging by the appearance of the region we may believe that straining of the tendon is not cured when there is only subcutaneous infiltration.—*Veterinary Journal.*

Operations in Hairy Regions. It is well to remember (*Int. Journ. Surg.*) that it is practically impossible to asepticize hair.

Hence, operations or treatment of wounds upon hairy parts, and especially the scalp, should be preceded by the shaving of an ample area. Patients often object to the removal of much hair from the scalp, but a little talk about the dangers of gangrene of this part will usually overcome their objection. [The above advice will aptly apply to veterinary practice. During the past year we have made it a rule to shave an ample area around the seat of an operation or wound, and the results have been in every way eminently satisfactory. Very few owners will offer any serious objections to this practice, when the benefit from so doing is pointed out to them.—W. J. M.]

